# Paeoniflorin Inhibits Migration and Invasion of Human Glioblastoma Cells via Suppression Transforming Growth Factor β-Induced Epithelial–Mesenchymal Transition

**DOI:** 10.1007/s11064-018-2478-y

**Published:** 2018-02-08

**Authors:** Zhaotao Wang, Zhi Liu, Guoyong Yu, Xiaohu Nie, Weiqiang Jia, Ru-en Liu, Ruxiang Xu

**Affiliations:** 1 Affiliated Bayi Brain Hospital, General Army Hospital, Southern Medical University, Beijing, People’s Republic of China; 2 Department of Neurosurgery, Peking University People’s Hospital, Peking University, Beijing, People’s Republic of China; 3 Nanchang University Medical College, Nanchang, Jiangxi People’s Republic of China; 4 Huzhou Central Hospital, Zhejiang, People’s Republic of China

**Keywords:** Paeoniflorin, Glioblastoma, EMT, Migration and invasion, TGFβ

## Abstract

Paeoniflorin (PF) is a polyphenolic compound derived from Radix Paeoniae Alba thathas anti-cancer activities in a variety of human malignancies including glioblastoma. However, the underlying mechanisms have not been fully elucidated. Epithelial to mesenchymal transition (EMT), characterized as losing cell polarity, plays an essential role in tumor invasion and metastasis. TGFβ, a key member of transforming growth factors, has been demonstrated to contribute to glioblastoma aggressiveness through inducing EMT. Therefore, the present studies aim to investigate whether PF suppresses the expression of TGFβ and inhibits EMT that plays an important role in anti-glioblastoma. We found that PF dose-dependently downregulates the expression of TGFβ, enhances apoptosis, reduces cell proliferation, migration and invasion in three human glioblastoma cell lines (U87, U251, T98G). These effects are enhanced in TGFβ siRNA treated cells and abolished in cells transfected with TGFβ lentiviruses. In addition, other EMT markers such as snail, vimentin and N-cadherin were suppressed by PF in these cell lines and in BALB/c nude mice injected with U87 cells. The expression of MMP2/9, EMT markers, are also dose-dependently reduced in PF treated cells and in U87 xenograft mouse model. Moreover, the tumor sizes are reduced by PF treatment while there is no change in body weight. These results indicate that PF is a potential novel drug target for the treatment of glioblastoma by suppression of TGFβ signaling pathway and inhibition of EMT.

**Electronic supplementary material:**

The online version of this article (10.1007/s11064-018-2478-y) contains supplementary material, which is available to authorized users.

## Introduction

Malignant gliomas are the most common and deadly brain tumors. Glioblastoma, characterized as rapid growth and highly invasiveness, is the most malignant in all glioma pathological types [1–3]. Over the past decades, although a variety of therapeutic approaches have been developed, including surgery, chemotherapy, radiotherapy or combined modalities, the average survival time of patients diagnosed with glioblastoma is seldom more than 15 months [4, 5]. Thus, it is urgent to discover new agents to cure the glioblastoma.

According the statistics from Food and Drug Administration (FDA), about 25–48% of current approved anti-cancer agents are derived from plants [6, 7]. In addition, new anticancer drugs from natural compounds are still being found every year [8, 9]. Therefore, natural compounds could be considered as a potential source of new anticancer drugs to resist glioblastoma.

Paeoniflorin (PF), as a traditional Chinese herbal medicine and a monoterpene glucoside natural compound, is the major active ingredient of *Paeonia lactiflora* Pall, PF has been previously studied mainly in anti-inflammation, antioxidant, neuroprotection and metabolic regulation [10–14], but increasing number of investigations indicate that PF exhibits anticancer activity. The underlying mechanisms have been studied, including that PF induces apoptosis, and have anti-proliferation, anti-metastasis, and anti-invasion effects to tumor cells. PF inhibited proliferation and invasion through suppressing Notch-1 signaling pathway in breast cancer cells [15], and inhibited human gastric carcinoma cell proliferation through up-regulation of microRNA-124 and suppression of PI3K/Akt and STAT3 signaling [16]. PF is also reported to inhibit the tumor invasion and metastasis in human hepatocellular carcinoma cells [17]. Recently, Xiao et al. reported that PF could potentiate the inhibitory effects of Erlotinib in pancreatic cancer by reducing ErbB3 phosphorylation [18]. Moreover, it has been reported that PF inhibited proliferation and induced apoptosis of human glioma cells via upregulating microRNA-16 and downregulating matrix metalloproteinase-9 (MMP9) [19]. Furthermore, in our previous study, we reported that PF inhibited human glioma cells via downregulating STAT3 [20]. Though several investigations have explored PF-mediated anticancer function, the underlying mechanisms are not fully clarified in glioblastoma.

Epithelial-to-mesenchymal transition (EMT), characterized by the loss of cell-to-cell adhesion, has been reported play a pivot role in tumor progression and metastasis in diverse solid tumors [21–24]. Once EMT process is activated, tumor cells acquire an invasive capacity that allows to invade ambient tissues and blood vessels and/or detach from the primary site [25, 26]. Though it is controversial about the EMT of glioblastoma, in the neuro-epithelial context, an increasing number of evidence has confirmed the existence of EMT-like process in glioblastoma. Activation of glioblastoma EMT-like program has been proved to promote the malignant progress, involving migration and invasion in vitro and in vivo [27–30]. It is likely to suppress initiation and progress of EMT could effectively inhibit glioblastoma. EMT regulation involves various molecules and signaling pathways. Transforming growth factor-beta (TGFβ), as a crucial cytokine and a member of transforming growth factors, has been demonstrated to play an important role in regulation of EMT. Rafehi et al. reported that TGFβ could regulate epithelial–mesenchymal plasticity in ovarian cancer ascites-derived spheroids [31]. And Shao et al. reported TGFβ could induce EMT in neuroblastoma cells [32]. Moreover, endogenous expression of TGFβ is high in glioblastoma, and some studies demonstrated that therapy targeting TGFβ-induced EMT could inhibit glioblastoma growth [33–35]. Therefore, whether TGFβ inactivation that inhibits EMT can prevent the onset and progression of glioblastoma is a considerable new potential approach in glioblastoma treatment.

In the present study, we examined the effects of PF on cell proliferation, apoptosis, migration and invasion in human glioblastoma cell lines. We further explored whether these effects are due to regulation of EMT via modulation of TGFβ expression and activity by PF in glioblastoma. In addition, we confirmed these findings by overexpression TGFβ using lentiviruses and knockdown of TGFβ using TGFβ siRNA in human glioblastoma cells. Furthermore, we examined whether PF suppresses tumor growth in U87 xenograft mouse model, and tested effects of PF on expression of TGFβ and its downstream MMP2/9, as well as the EMT markers.

## Method and Materials

### Chemicals, Reagents and Antibodies

PF was purchased from Tianjin Shilan Science and Technology Ltd (Tianjin, China). PF was dissolved in normal saline and stored at 4 °C. Dulbecco’s modified Eagle’s medium (DMEM) and fetal bovine serum (FBS) were purchased from Gibco (Grand Island, USA). Antibodies against TGFβ, MMP2, vimentin, GAPDH were purchased from Cell Signaling Technology (Beverly, MA). Antibodies against MMP9 and snail were purchased from Abcam (Cambridge, MA).

### Cell Culture

The human glioblastoma cell lines U87, U251, T98G were purchased from Chinese Academy of Medical Sciences (Beijing, China). These cell lines were cultured in Dulbecco’s modified Eagle’s medium (DMEM) supplemented with 10% fetal bovine serum (FBS) and incubated at 37 °C in a humidified atmosphere in 5% CO_2_.

### Cell Viability Assay

Cells were seeded at 4 × 10^3^ cells/well in a 96-well plate for 24 h and treated with different concentrations of PF. After 24 h, 10 µl of the CCK-8 solution was added to each well and incubated for 1 h at 37 °C. Then, the reaction mixture was measured by the microplate.

### Cell Apoptosis Analysis

Cells were cultured in a 6-well plate overnight and treated with various concentrations of PF for 24 h. Then, cells were harvested and washed with phosphate buffer saline (PBS), resuspended in 500 µl binding buffer with 5 µl propidium iodide (PI) and 5 µl FITC-conjugated anti-Annexin V antibody. Apoptosis was analyzed with an Accui C6 flow cytometer (BD, USA).

### Wound Healing Assay

A wound-healing assay was used to compare the migratory ability of glioblastoma cells in control and experiment groups. Cells (5 × 10^5^ cells) were seeded and cultured into the 6-well plates. When the cells reached 80–90% confluence, similar size of scratches were introduced into the monolayer by a sterile pipette tip. The monolayer cells were rinsed with PBS to remove detached cells, and then replaced with medium containing various concentration of PF or normal saline. To discriminate the contributions of cell proliferation and migration to wound closure, cell cycle blocker hydroxyurea (5 mM, Sigma, Aldrich) was added at the time of the experiment. To analyze the cell migration, the wounded areas were photographed at the indicated time points with Leica microscope (Melville, NY) and processed using image pro plus software (NIH). Percentage of wound healing was measured as following: [1 − (empty area × h/empty area 0 h)] × 100.

### Cell Invasion Assay

The transwell system for assay of cell invasion was obtained from Corning (Corning, USA). Cells (1 × 10^5^ in 200 µl DMEM supplemented with 1% FBS) were seeded in the upper chamber (8 µm) coated with 100 µl matrigel (BD Biosciences, CA, USA). The lower chamber was filled with 600 µl DMEM supplemented with 20% FBS and indicated concentrations of PF. After 24 h, the cells in the lower chamber were fixed by methanol, stained with 0.1% crystal violet in methanol, and photographed in three independent 100 × fields for each well. Then the cells of every field were counted.

### Transfection

To overexpression TGFβ, glioblastoma cell lines were transfected with lentiviral vector carrying TGFβ–eGFP or eGFP only (GeneCopoeia, Maryland Rockville, USA). Puromycin was applied to obtain stable transfected cells. To knockdown of TGFβ, glioblastoma cell lines were transfected with TGFβ siRNA or empty vector using lipofectamine 3000 following the manufactory protocol. TGFβ siRNA: sense 5ʹ-GATGCCTACACAGGTGTGTAT-3ʹ; antisense 3ʹ-GCAGACTAGACTACGGTTCAA-5ʹ.

### RNA Preparation and Real-Time Polymerase Chain Reaction

Total RNA was isolated using an E.Z.N.A. Total RNA Kit (Omega Bio-Tek, Norcross, GA, USA). The cDNA was reverse transcripted from 1 µg of total RNA using a Prime-Script II 1st Strand cDNA Synthesis Kit (Takara,Shiga, Japan). Gene expression was determined by real-time polymerase chain reaction (PCR) using a SYBR Premix Ex Taq Kit (Takara) and an ABI Vii7 detection system (Applied Biosystems, Kumamoto, Japan). The sequences of PCR primers used in this study are listed in Table S1.

### Western Blotting

Western blots were performed using glioblastoma cell lysates or xenograft glioblastoma tissue homogenates. Protein was extracted using Pro-prep TM protein Extraction Solution (iNtRON Biotechnology, Korea) according to manufacturer’s instructions. Equal amounts of total protein were separated on 10–12% sodium dodecyl sulfate-polyacrylamide gel electrophoresis (SDS-PAGE), transferred to polyvinylidene difluoride membranes (Merck, KGaA, Darmstadt, Germany). The membranes were blocked with 5% BSA at room temperature for 1 h, and then incubated with specific primary antibodies overnight at 4 °C. The appropriate secondary antibodies conjugated with HRP were incubated for 1 h at room temperature, signal was obtained using Super Signal ECL (Pierce, Rockford, IL, USA).

### U87 Xenograft Mouse Model Paeoniflorin Treatment

Female BALB/c nude mice were obtained from Vital River Laboratories (Beijing, China). Mice were aged 6–8 weeks and kept under a standard protocol approved by the Institutional Animal Care of Army General Hospital. All procedures performed in studies involving animals were in accordance with the ethical standards of the institution or practice at which the studies were conducted. Each mouse was injected subcutaneously with cultured U87 cells (5 × 10^6^ cells per mouse) into the dorsum. The tumor size was measured in two orthogonal directions using calipers, and the tumor volume (mm^3^) was calculated using the equation: 1/2 × length × width^2^. When the tumors grew to about 150 mm^3^, the tumor–bearing mice were distributed into two groups (n = 5 each) and orally fed with PF (1 g/kg/day) or vehicle (equivalent amount of PBS) Tumor sizes and body weights were measured once every four days. At the end of these experiments, the mice were sacrificed and the tumors were resected and homogenized for western blotting.

### Statistical Analysis

The data are presented as the mean ± standard deviation from at least three independent experiments. Simple comparisons between two groups were analyzed using independent t-tests. Multiple comparisons between the groups were performed using one-way ANOVA followed by post-hoc analysis with LSD or Dunnett’s T3 test on SPSS 20.0 software. P < 0.05 was considered statistically significant.

## Results

### Effects of PF on Cell Proliferation, Apoptosis, Migration and Invasion in Glioblastoma Cells

**Fig. 1:**
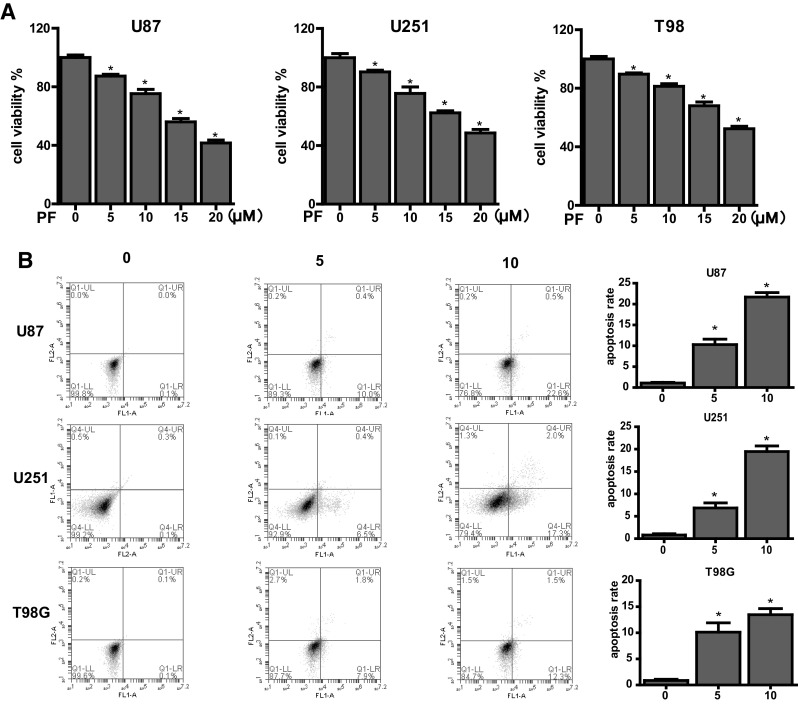
Effects of paeoniflorin (PF) on proliferation, apoptosis in U87, U251, T98 cells. **a** Cells were incubated with the indicated concentrations of PF for 24 h before CCK-8 assay. **b** Cell apoptosis in glioblastoma cells treated with PF was determined by flow cytometry. Each treatment was replicated at least three times. All tests were performed in triplicate and presented as mean ± standard error. *P < 0.05, compared with control (0 µM)

To investigate the effects of PF on cell proliferation, we performed the CCK-8 experiments. As shown in Fig. 1a, 24-h PF treatment significantly inhibited cell growth in U87, U251 and T98G glioblastoma cell lines in a dose-dependent manner. Cell viability was declined from 90 to 80% in all three cell lines when concentration of PF was increased from 5 to 10 µM.

We next aimed to examine if cell growth inhibition by PF due to PF-induced cell apoptosis. PI-FITC-annexin assay was used to evaluate the cell apoptosis in U87, U251 and T98G treated with 5 and 10 µM PF for 24 h. As presented in Fig. 1b, PF significantly triggered cell apoptosis in all three glioblastoma cells (Fig. 1b). There was only 1% cell death in 24 h under normal condition without PF treatment in all three cells. However, percentage of cell death was increased from 10 to 22, 7 to 19 and 10 to 14% in U87, U251, T98G cells, respectively, when increased PF concentration from 5 to 10 µM. Our results demonstrate that PF dose-dependently trigger cell death and are consistent with the previous studies indicating PF induced apoptosis in U87 and U251 glioblastoma cells.

**Fig. 2:**
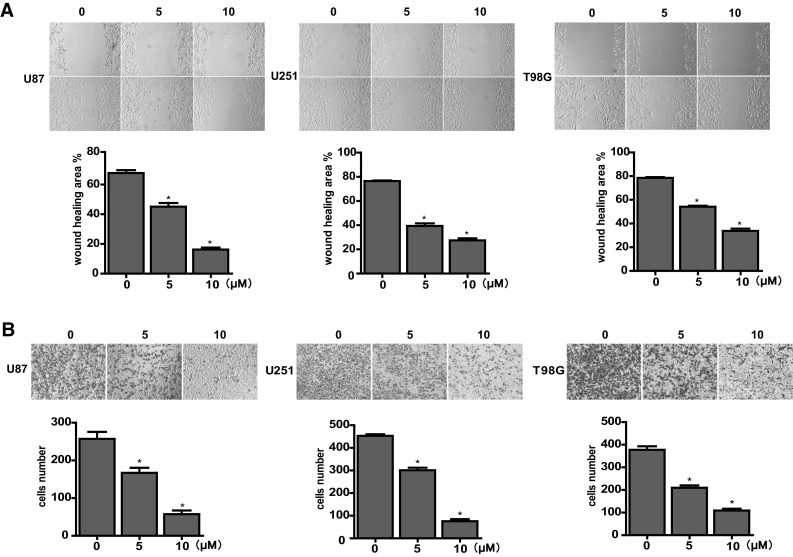
Effects of paeoniflorin (PF) on migration and invasion in U87, U251, T98 cells. **a** Cells were treated with 0, 5 or 10 µM PF after the wounds were scratched. Then representative images of wound healing were acquired after 0 or 24 h. Percentage of wound healing was measured via image-Pro Plus software then was calculated through the formula: [1 − (empty area × h/empty area 0 h)] × 100. **b** Cells were incubated with the indicated concentrations of PF for 24 h followed by methyl alcohol fixation and crystal violet staining. Then representative images of cells that invaded into the bottom of the membrane were obtained. The stained cells were counted. Each treatment was replicated at least three times. All tests were performed in triplicate and presented as mean ± standard error. *P < 0.05, compared with control (0 µM)

We also conducted wound healing assay and cell invasion assay to evaluate the effects of PF on glioblastoma cell migration and invasion ability. Low doses PF (5 and 10 µM) were used in control and experimental groups to prevent the effects of PF on induction of cell death. Compared with the untreated groups, the PF treated groups exhibit less cells migrating into the wounds in association with less cells invading into the bottom of the insert membranes (Fig. 2a, b). These results demonstrate that PF significantly inhibits glioblastoma cell migration and invasion.

### Effects of PF on Expression of TGFβ, EMT Markers and MMP2/9 in Glioblastoma Cells

**Fig. 3:**
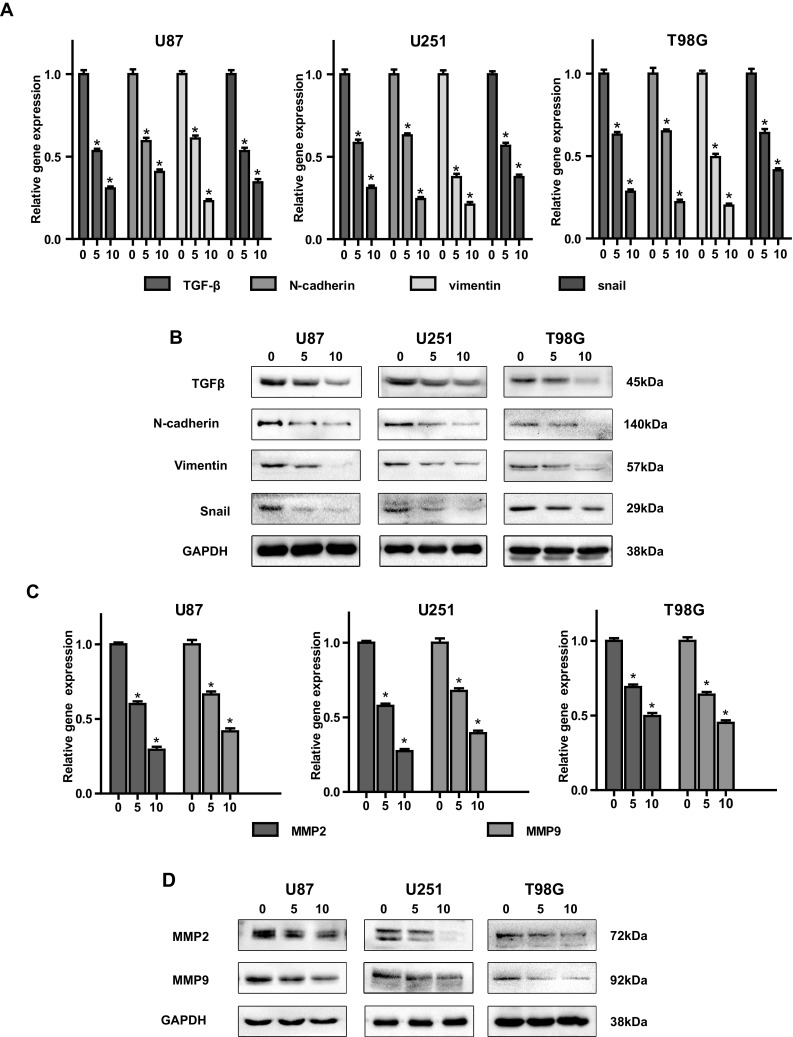
Paeoniflorin (PF) downregulated TGFβ, mesenchymal markers and MMP2/9 in glioblastoma cells. **a** Expression of TGFβ and EMT makers was examined by real-time PCR after indicated concentration of PF treatment for 12 h. **b** The protein expression of TGFβ and EMT makers was examined by western blotting after indicated concentration of paeoniflorin treatment for 24 h. **c** MMP2/9 mRNA expression was detected by real-time PCR after 0, 5, 10 µM paeoniflorin treatment for 12 h. **d** MMP2/9 protein expression was evaluated by western blotting after 0, 5, 10 µM paeoniflorin treatment for 24 h. All tests were replicated at least three times and each image represented at least three independent results. *P < 0.05, compared with control (0 µM)

TGFβ has been reported to be an oncoprotein in glioblastoma [34]. Therefore, inhibition of TGFβ could be a potential effective way for cure glioblastoma. EMT process contributes to glioblastoma progress [36], suppression of EMT process is a promising approach to treat glioblastoma. We next investigated whether PF can regulate the expression of TGFβ and EMT makers. Our results demonstrated that PF dose-dependently reduced the expression of TGFβ, snail, N-cadherin, vimentin and MMp2/9 at both mRNA and protein levels (Fig. 3a–d). These results indicated that PF plays a critical role in regulation of TGFβ, and TGFβ-induced EMT in glioblastoma.

### Overexpression of TGF-β Abolishes the Effects of PF in Glioblastoma Cells

**Fig. 4:**
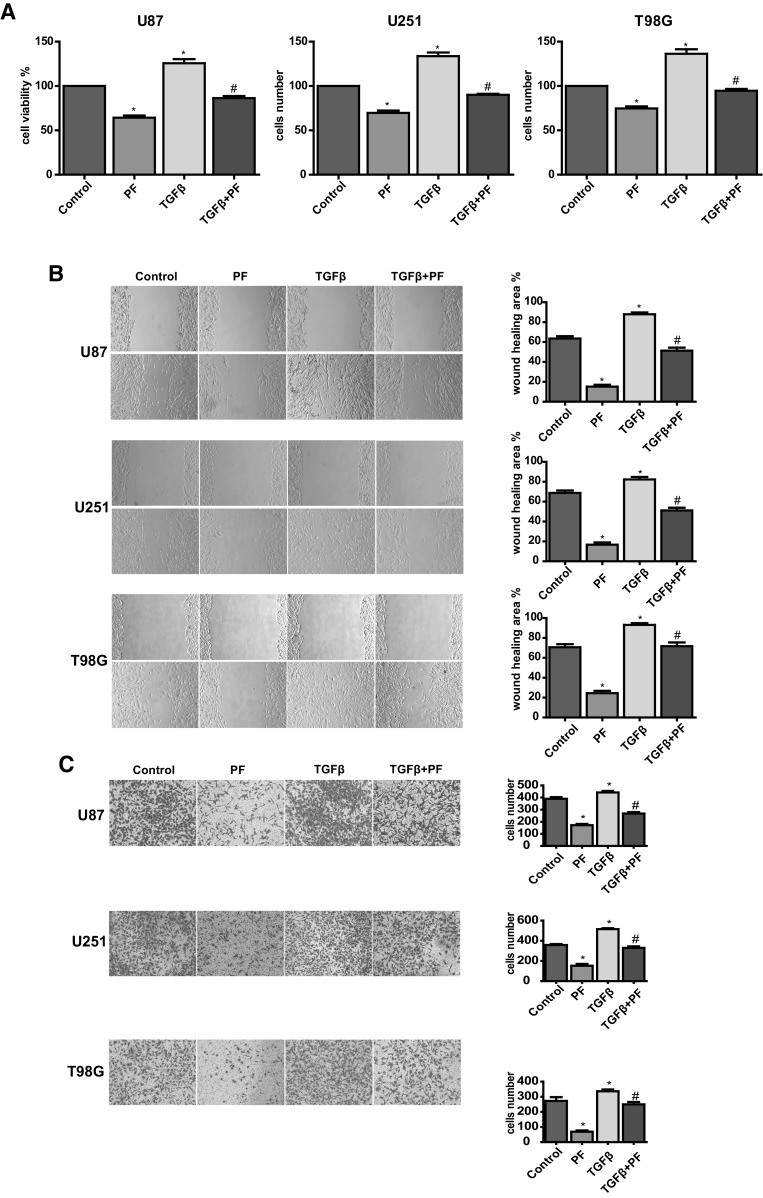
Over-expression of TGFβ rescues paeoniflorin-induced cell proliferation, migration and inhibition. **a** Effect of TGFβ overexpression on paeoniflorin treated U87, U251 and T98G cell proliferation. **b** (top panel) Effect of TGFβ overexpression on paeoniflorin treated U87, U251 and T98G cell migration. **c** Effect of TGFβ overexpression on paeoniflorin treated U87, U251 and T98G cell invasion. Control: GFP lentivirus transfection; PF: GFP lentivirus transfection + 10 µM paeoniflorin; TGFβ: lentivirus transfection TGFβ; TGFβ + PF: lentivirus transfection TGFβ + 10 µM paeoniflorin. *P < 0.05 versus control. ^#^P < 0.05, compared with either paeoniflorin treatment or TGFβ transfection alone

To further investigate whether PF causes anti-EMT effects by inhibition of TGFβ in glioblastoma cells, we generated stable transfected U87, U251 and T98G cell lines overexpressing TGFβ by transfection of a lentiviral vector carrying eGFP tagged TGFβ cDNA and selected by puromycin. Cells with eGFP stable transfection were used as TGFβ negative controls. The TGFβ-overexpressing cells were incubated with PF for 24 h. We found that overexpression of TGFβ increased tumor cell proliferation (Fig. 4a), and it was normalized by PF treatment in all three glioblastoma cells (Fig. 4a).

**Fig. 5:**
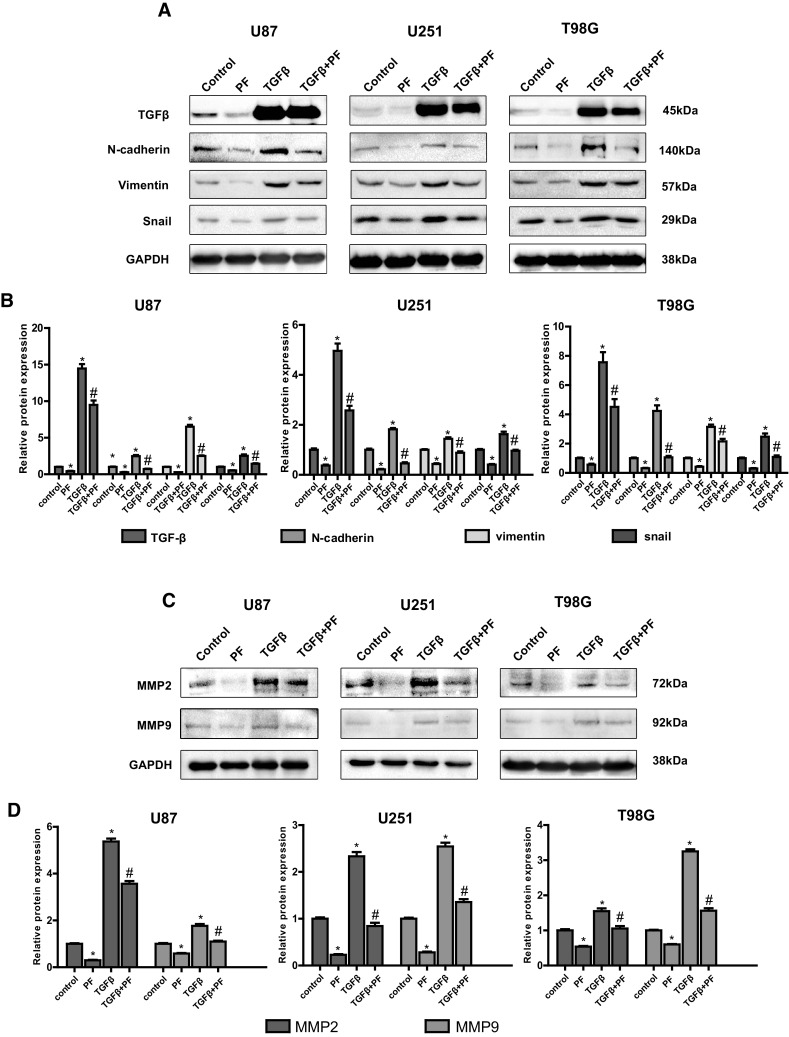
Over-expression of TGFβ rescues paeoniflorin-induced TGFβ, EMT makers and MMP2/9 downregulation. **a** The protein expression of TGFβ and EMT makers was examined by western blotting with lentivirus transfection and paeoniflorin treatment. **b** Quantitative results of panel **a. c** MMP2/9 protein expression was evaluated by western blotting with lentivirus transfection and paeoniflorin treatment. **d** Quantitative results of panel **c**. Control: GFP lentivirus transfection; PF: GFP lentivirus transfection + 10 µM paeoniflorin; TGFβ: lentivirus transfection TGFβ; TGFβ + PF: lentivirus transfection TGFβ + 10 µM paeoniflorin. *P < 0.05 versus control. ^#^P < 0.05, compared with either paeoniflorin treatment or TGFβ transfection alone

We also found that upregulation of TGFβ enhanced glioblastoma cell migration (Fig. 4b) and invasion (Fig. 4c). Both glioblastoma cell migration and invasion promoted by overexpression of TGFβ were abolished by treatment with PF. Upregulation of TGFβ expression in stable transfected tumor cell lines were validated by western blotting (Fig. 5a, b). In addition, we also observed that downstream EMT makers: snail, N-cadherin, vimentin, and MMP2/9 were induced by upregulation of TGFβ in a dose-dependent manner, and were normalized with low dose of PF treatment (Fig. 5a–d). These results suggest that PF plays an anticancer role, at least, partly via down-regulation of TGFβ-induce EMT in glioblastoma cells.

### Down-Regulation of TGF-β Enhances the Effects of PF in Glioblastoma Cells

**Fig. 6:**
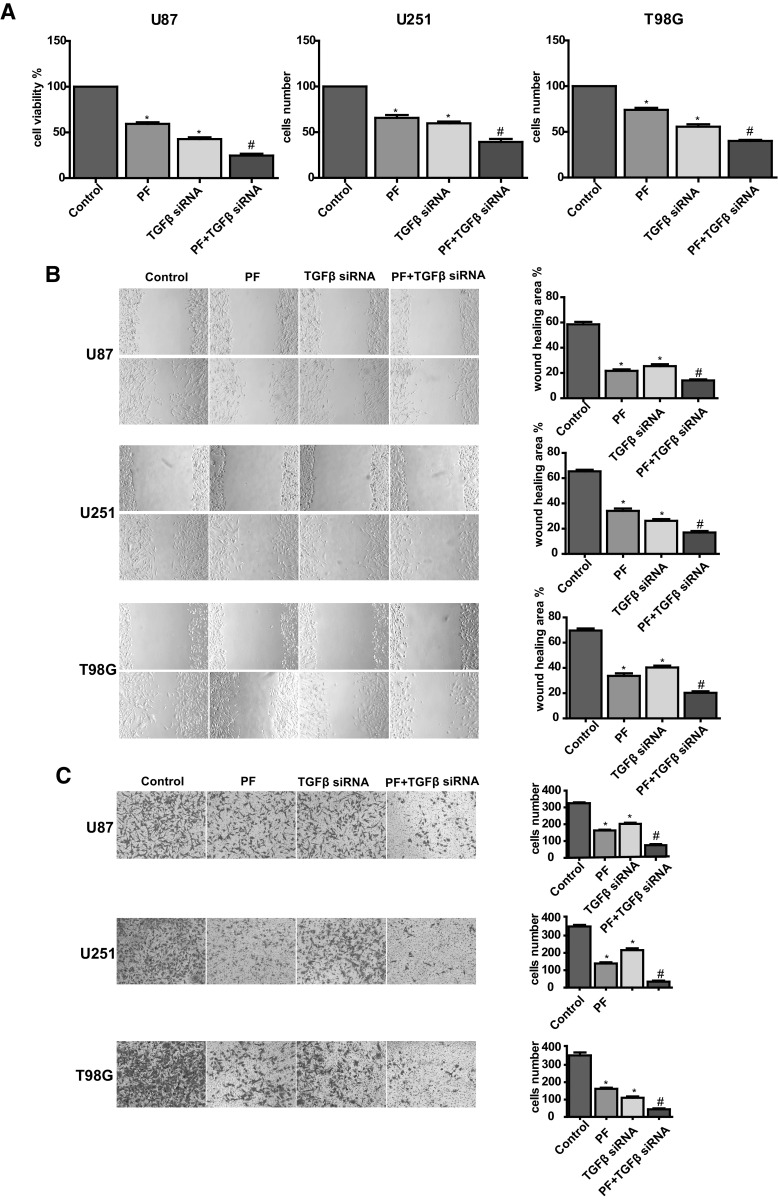
TGFβ knockdown enhances paeoniflorin-induced cell proliferation, migration and inhibition. **a** Effect of TGFβ knockdown on paeoniflorin treated U87, U251 and T98G cell proliferation. **b** (top panel) Effect of TGFβ knockdown on paeoniflorin treated U87, U251 and T98G cell migration. (bottom panel) Quantitative results are illustrated for top panel. **c** (top panel) Effect of TGFβ knockdown on paeoniflorin treated U87, U251 and T98G cell invasion. bottom panel, quantitative results are illustrated for top panel. Control: scramble siRNA; PF: scramble siRNA + 10 µM paeoniflorin; TGFβ siRNA: TGFβ siRNA; TGFβ siRNA + PF: TGFβ siRNA + 10 µM paeoniflorin. *P < 0.05 versus control. ^#^P < 0.05, compared with either paeoniflorin treatment or TGFβ siRNA transfection alone

**Fig. 7:**
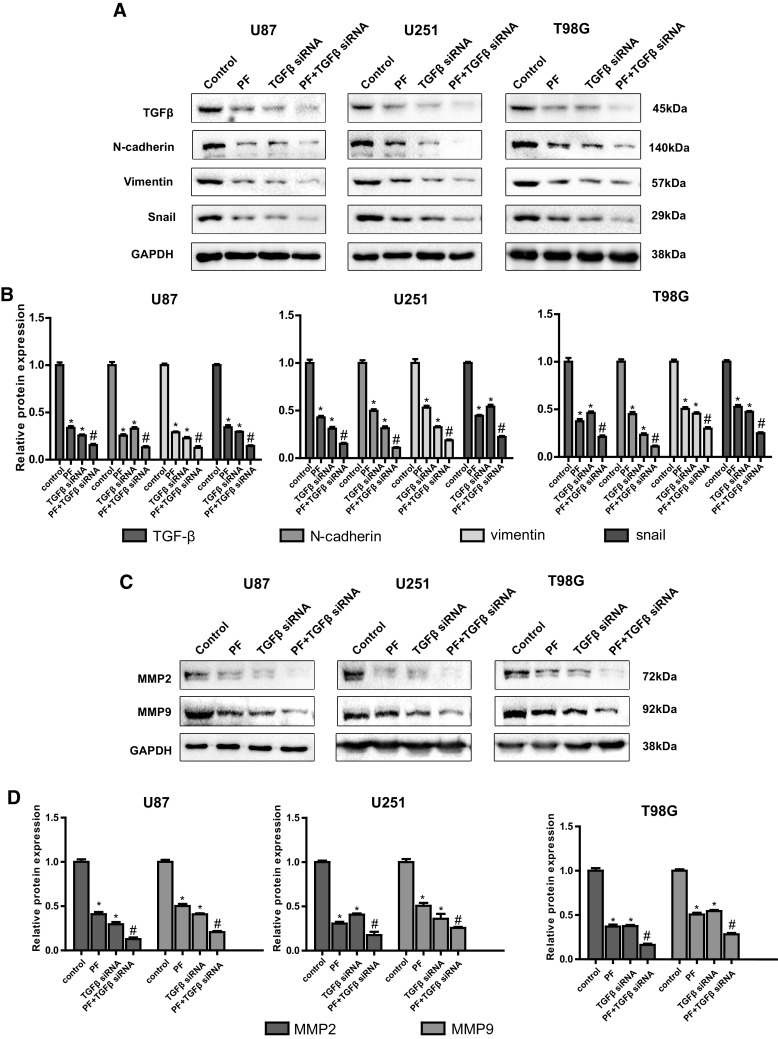
TGFβ knockdown enhances paeoniflorin-induced TGFβ, EMT makers and MMP2/9 downregulation. **a** The protein expression of TGFβ and EMT makers was examined by western blotting with siRNA transfection and paeoniflorin treatment. **b** Quantitative results of panel **a. c** MMP2/9 protein expression was evaluated by western blotting with siRNA transfection and paeoniflorin treatment. **d** Quantitative results of panel **c**. Control: scramble siRNA; PF: scramble siRNA + 10 µM paeoniflorin; TGFβ siRNA: TGFβ siRNA; TGFβ siRNA + PF: TGFβ siRNA + 10 µM paeoniflorin. *P < 0.05 versus control. ^#^P < 0.05, compared with either paeoniflorin treatment or TGFβ siRNA transfection alone

To further confirm the oncogenic role of TGFβ on PF-mediated anticancer effect, TGFβ was knockdown using a specific siRNA in glioblastoma cells. We found that knockdown of TGFβ inhibited cell proliferation in U87, U251 and T98G cells (Fig. 6a). Cells treated with TGFβ siRNA or PF both exerted potent suppression of cell growth (Fig. 6a), inhibition of cell migration (Fig. 6b) and invasion (Fig. 6c), and combination treatments exhibited a significant greater extent effect than applied with TGFβ siRNA and PF alone. Down-regulation of TGFβ expression in siRNA transfected tumor cell lines were validated by western blotting (Fig. 7a, b). Moreover, our results also demonstrated that snail, N-cadherin, vimentin, and MMP2/9 were inhibited by down-regulation of TGFβ in a dose-dependent manner, and were further suppressed with low dose of PF treatment (Fig. 7a–d). These results further verified that PF exerted an anticancer function partly through down-regulation of TGFβ expression and diminished TGFβ-induced EMT in glioblastoma cells.

### PF Suppresses Glioblastoma Growth in a U87 Xenograft Mouse Model

**Fig. 8:**
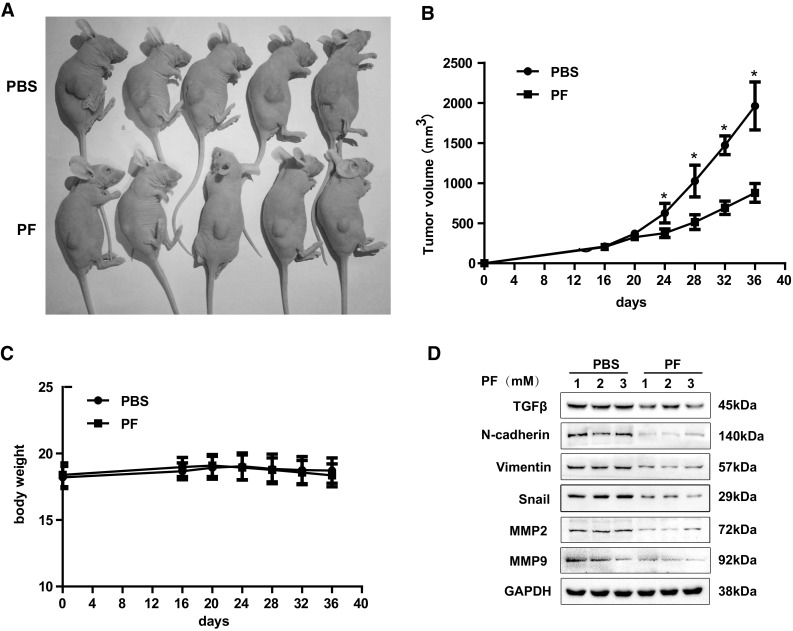
Effects of paeoniflorin (PF) in xenograft mouse models. **a** Inhibition in the size of the xenograted U87 tumors were photographed. The tumor volume (**b**) and body weight (**c**) were measured per 4 days. **d** At the end of the experiments, tumor tissues were excised from the mice, and then the protein lysates were analyzed by western blot analysis. Each western blotting image represented at least three independent results

We further explored the inhibitory effects of PF on glioblastoma growth in a U87 xenograft mouse model. The results presented in Fig. 8 that tumor volumes were significantly decreased (Fig. 8a, b), but no changes in body weights (Fig. 8c) in 24 days PF-treated group in comparison with vehicle-treated group. We confirmed that the endogenous TGFβ expression, snail, N-cadherin, vimentin, and MMP2/9 in tumors dissected from the U87 xenograft mice was dose-dependently suppressed by PF (Fig. 8d). Our results from in vivo studies are consistent with in vitro results demonstrating that PF may play a critical role on suppression of glioblastoma growth via inhibition of TGFβ-induced EMT.

## Discussion

Cell migration and invasion are important tumor progression process, promoting the glioblastoma cells to invade and infiltrate into the surrounding normal brain tissue which leads it difficult to remove completely and the epibiotic glioblastoma after surgery frequently causes relapse [5]. Ways that specifically suppress or reverse these malignant features have been proven to be valuable in some basic and preclinical studies [4]. Hence, identifying novel therapeutic approaches that inhibit migration and invasion are key elements in effectively curing glioblastoma. In this study, we reported that PF successfully inhibited migration and invasion in glioblastoma.

Natural compounds emerge as an alternative resource for new antitumor drug discovery, and there is an enhanced interest in seeking new effective agents for the treatment of glioblastoma from natural compounds. PF, as a monomeric natural compound extracted from *Radix paeonia Alba*, has showed a variety of biological activities such as anti-oxidation, anti-inflammation, neuroprotection, immunoregulation, etc [10, 12, 13, 37]. In addition, a few studied have been done to investigate anti-cancer activity as well as the underlying mechanisms of PF, which showed that PF could inhibit some tumor types though inducing apoptosis, cell cycle arresting involving multiple signaling molecules like NF-kB, stat3, Notch1 or p53 pathway [15, 38, 39]. In our study, we found that PF could inhibit migration and invasion, involving the suppression of TGFβ-mediated EMT.

EMT, as a documented mechanism during tumor progression, can promote tumor growth through enhancing the ability of invasion, increasing drug resistance and sustaining stemness in tumor stem cells [40–42]. Though existing controversial in EMT of glioblastoma, based on the neuroepithelial context, it has been demonstrated that members of the TWIST- and SNAL-family, both established groups of EMT-activators, do enhance GBM-cell motility and invasiveness both in vitro and in vivo as shown in animal studies and in patient-derived specimens. In this respect, the recently defined mesenchymal subgroup of GBMs glioblastoma can acquire the characteristics of epithelial phenotype [27, 28, 30, 43]. It has been reported that glioma suppression of EMT process inhibited glioblastoma. Moreover, it reported that PF could prevent hypoxia-induced epithelial–mesenchymal transition in human breast cancer cells [37]. In our study, PF showed anti-migration and invasion activity through reversing the EMT progress, which suggests PF maybe another candidate to suppress EMT in glioblastoma.

The MMP family plays a pivotal role in the degradation of extracellular matrix (ECM) in diverse physiological and pathological situation. Emerging evidence has suggested that MMPs facilitated cancer cell invasion into the surrounding normal tissues via the cell-surface ECM degradation [44, 45]. There is convincing relationship between raised MMP levels and tumor cell invasiveness in human glioblastoma. Among them, more attention has been concentrate on MMP-2 and MMP-9. MMP-9 level was intensely associated with glioblastoma grade [46]. Some studies also indicated that MMP-2 was correlated with glioblastoma invasion, angiogenesis, metastasis, and relapse [47]. Additionally, suppression of MMP-2 and MMP successfully inhibited or delayed glioblastoma invasion and migration in vitro and in vivo [48]. Moreover, it exists crosstalk between EMT and MMPs, which can regulate the invasion and migration in glioblastoma cells. Overexpression of MMP-2 or MMP-9 strengthens EMT process in glioblastoma [49, 50]. Likewise, the EMT process could induce MMPs expression, suggesting the positive feedback loop between MMPs and EMT synergistically facilitates the migration and invasion in malignant glioblastoma. As our results showed, PF exerted anti-migration and invasion via inhibiting the EMT and MMP2/9, which reflects the dual suppressive effects of PF in glioblastoma.

TGFβ, as a multifunctional cytokine that has been demonstrated as a regulator in glioblastoma initiation and progression because of its effects on tumor invasion, cell proliferation, angiogenesis, immunosuppression and the maintenance of stemness of glioblastoma stem cells (GSCs) [29, 36, 51]. In addition, TGFβ is overexpressed in glioblastoma but not in normal brain tissues, further implying that TGF-β prompts glioblastoma development [33]. Though TGFβ play a suppressive role in the initial stage of glioblastoma, but it will improve the exacerbation of glioblastoma in evolved stage [52]. Thus, targeting TGFβ have been a strategy to cure glioblastoma. Besides, an increasing number of natural compounds have been found, which can suppress EMT via inhibiting TGFβ in tumor. Decitabine can reverse TGF-β-induced EMT in non-small-cell lung [53]. Baicalin could inhibits human osteosarcoma cells invasion by suppressing TGFβ-induced EMT [54]. Calycosin could inhibit migration and invasion through suppressing TGFβ-mediated EMT in U87 and U251 cells [55]. In our study, PF decreases TGFβ in protein level, but when forced TGFβ, the glioblastoma begins to become resisted to PF of TGFβ level as well as migration and invasion, suggesting that suggesting that PF, at least partially, can target TGFβ. Following, the downstream molecules about EMT and MMP2/9 also been partially reversed, which implies the proliferation target TGFβ to regulate EMT and MMP2/9.

To meet the complex research needs, various glioblastoma cells have been established, which has diverse gene mutation background. Also in clinical, glioblastomas from different patients may have multiple gene mutation, so it is urged to find a reagent that can play an extensive role in suppressing glioblastoma. In our study, we used three glioblastoma cells lines U87, U251, T98G to verify our results. Among them, T98G is overexpressed O6-methylguanine-DNA methyltransferase (MGMT), which is widely accepted mechanism to resist temozolomide (TMZ) [56, 57], but eventually PF still showed strongly suppression in T98G, which suggests that PF can exert wide suppression function in glioblastoma.

Though a growing number of natural compounds have displayed anti-cancer activities in vitro but among them only a few can play the anti-cancer role in vivo [6, 9]. As for PF, previous studies has showed it could be well tolerated in vivo [10, 12].Moreover, PF does have the capability to cross the blood–brain barrier. For example, He et al. found that PF could quickly penetrate through blood–brain barrier (BBB) to reach hippocampus and maintain a high concentration [58]. Similarly, Cao et al. reported that paeoniflorin could penetrate through the blood–brain barrier to reach the normal cortex and could reach the effective concentration to treat ischemia–reperfusion rats [59]. And in our following study, we plan to investigate the effect of PF on orthotopic glioma model in mouse. And in our study, PF administration in oral a way exerted anti-cancer activities on established glioblastoma xenografts, which consists the results in vitro. Besides, there are no difference of weight between the mouse applying and without applying, verifying the mouse can well tolerate the drug.

In conclusion, we found that PF acts as a suppressor of cell migration and invasion in glioblastoma in vitro and vivo. What is more, we identify TGF-induced EMT process may be the therapeutic target of PF in glioblastoma. PF may be a potential natural compound to cure glioblastoma.

## Electronic supplementary material

Below is the link to the electronic supplementary material.

Supplementary material 1 (DOCX 62 KB)
