# Supplementary material for: Paeoniflorin Inhibits Migration and Invasion of Human Glioblastoma Cells via Suppression Transforming Growth Factor β-Induced Epithelial–Mesenchymal Transition
Source: Neurochem Res. 2018 Feb 8;43(3):760–74. doi: 10.1007/s11064-018-2478-y (PMC5842263; doi:10.1007/s11064-018-2478-y)
Supplement: Supplementary file 1 — Supplementary material 1 (DOCX 62 KB) [file 11064_2018_2478_MOESM1_ESM.docx]

**Table S1**

| Primers for quantitative real-time RT-PCR analysis of gene transcript expression | | | |
| --- | --- | --- | --- |
| Gene | Forward primer | Reverse primer | Size(bp) |
| TGFβ | CCCAGCATCTGCAAAGCTC | GTCAATGTACAGCTGCCGCA | 101 |
| MMP2 | CCCACTGCGGTTTTCTCGAAT | CAAAGGGGTATCCATCGCCAT | 89 |
| MMP9 | AGACCTGGGCAGATTCCAAAC | CGGCAAGTCTTCCGAGTAGT | 94 |
| CDH2 | GGACAGTTCCTGAGGGATCA | GGATTGCCTTCCATGTCTGT | 253 |
| VIM | AGTCCACTGAGTACCGGAGAC | CATTTCACGCATCTGGCGTTC | 98 |
| SNAI1 | TCGGAAGCCTAACTACAGCGA | AGATGAGCATTGGCAGCGAG | 140 |
| GAPDH | CTGGGCTACACTGAGCACC | AAGTGGTCGTTGAGGGCAATG | 101 |
